# Subinhibitory Concentrations of Antibiotics Exacerbate Staphylococcal Infection by Inducing Bacterial Virulence

**DOI:** 10.1128/spectrum.00640-22

**Published:** 2022-06-27

**Authors:** Peng Gao, Yuanxin Wei, Rachel Evelyn Wan, Ka Wing Wong, Ho Ting Venice Iu, Sherlock Shing Chiu Tai, Yongli Li, Hin Cheung Bill Yam, Pradeep Halebeedu Prakash, Jonathan Hon Kwan Chen, Pak Leung Ho, Kwok Yung Yuen, Julian Davies, Richard Yi Tsun Kao

**Affiliations:** a Department of Microbiology, Li Ka Shing Faculty of Medicine, The University of Hong Kong, Pok Fu Lam, Hong Kong; b Department of Microbiology, Queen Mary Hospital, Pok Fu Lam, Hong Kong; c State Key Laboratory of Emerging Infectious Diseases and the Research Centre of Infection and Immunology, Li Ka Shing Faculty of Medicine, The University of Hong Kong, Hong Kong; d Carol Yu Centre for Infection, The University of Hong Kong, Pok Fu Lam, Hong Kong; e Department of Microbiology and Immunology, The University of British Columbia, Vancouver, British Columbia, Canada; University Roma Tre

**Keywords:** *Staphylococcus aureus*, subinhibitory concentration, virulence, antibiotics

## Abstract

Antibiotics are widely used for the treatment of bacterial infections. However, injudicious use of antibiotics based on an empirical method may lead to the emergence of resistant strains. Despite appropriate administration of antibiotics, their concentrations may remain subinhibitory in the body, due to individual variations in tissue distribution and metabolism rates. This may promote bacterial virulence and complicate the treatment strategies. To investigate whether the administration of certain classes of antibiotics will induce bacterial virulence and worsen the infection under *in vivo* conditions. Different classes of antibiotics were tested *in vitro* for their ability to induce virulence in a methicillin-resistant S. aureus strain Mu3 and clinical isolates. Antibiotic-induced pathogenicity was assessed *in vivo* using mouse peritonitis and bacteremia models. *In vitro,* β-lactam antibiotics and tetracyclines induced the expression of multiple surface-associated virulence factors as well as the secretion of toxins. In peritonitis and bacteremia models, mice infected with MRSA and treated with ampicillin, ceftazidime, or tetracycline showed enhanced bacterial pathogenicity. The release of induced virulence factors *in vivo* was confirmed in a histological examination. Subinhibitory concentrations of antibiotics belonging to β-lactam and tetracycline aggravated infection by inducing staphylococcal virulence *in vivo*. Thus, when antibiotics are required, it is preferable to employ combination therapy and to initiate the appropriate treatment plan, following diagnosis. Our findings emphasize the risks associated with antibiotic-based therapy and underline the need for alternative therapeutic options.

**IMPORTANCE** Antibiotics are widely applied to treat infectious diseases. Empirically treatment with incorrect antibiotics, or even correct antibiotics always falls into subinhibitory concentrations, due to dosing, distribution, or secretion. In this study, we have systematically evaluated *in vitro* virulence induction effect of antibiotics and *in vivo* exacerbated infection. The major highlight of this work is to prove the β-lactam and tetracyclines antibiotics exacerbated disease is due to their induction effect on staphylococcal virulence. This phenomenon is common and suggests that if β-lactam antibiotics remain the first line of defense during empirical therapy, we either need to increase patient reliability or the treatment approach may improve in the future when paired with anti-virulence drugs.

## INTRODUCTION

The importance of timely administration of effective antibiotics in serious bacterial infections has been repeatedly emphasized (1). Therefore, antibiotics are often administered empirically to treat bacterial infections before antibiograms are available. Due to these factors, the administered antibiotics may not be effective against multidrug-resistant pathogens (2, 3). The microbiological effects of antibiotics extend beyond antibacterial activities. In nature, antibiotics function as signaling molecules (4). At subinhibitory concentrations, they modulate gene expression and alter bacterial physiology (5). Despite using “appropriate” antibiotics and dosages, their availability in the body remains lower than the MIC. This happens due to differences in tissue distribution and metabolic rates among individuals. As a result, ineffective doses of antibiotics may stimulate bacterial virulence and worsen the disease outcome (6). Voluminous research has shown that antibiotics at subinhibitory concentrations can increase S. aureus virulence. Among several classes of antibiotics, β-lactams have been reported to induce a plethora of virulence factors such as alpha-toxin, Panton–Valentine leukocidin (PVL), phenol-soluble modulins (PSMs), and capsule production *in vitro* (6–11). Although a recent *in vivo* study discovered S. aureus lipoprotein as a major factor in β-lactam induced pathogenesis, their findings mainly focused on hypercytokinemia (10). In contrast to these findings, the present study focused on the antibiotic-induced virulence expression in S. aureus and replicated the actual infections elicited by antibiotics *in vivo*.

We first employed ampicillin as an example to confirm its induction effect on S. aureus virulence factors expression and production *in vitro* at subinhibitory concentrations. Later, we used mouse models to show that β-lactam and tetracycline antibiotics, when used at clinically relevant concentrations, may worsen bacterial infection *in vivo*. From these findings we demonstrated that antibiotics at subinhibitory concentrations may enhance S. aureus pathogenicity and put forward the need for alternative therapeutics.

## RESULTS

### Subinhibitory concentrations of ampicillin enhanced S. aureus virulence factors expression and production *in vitro*.

**FIG 1:**
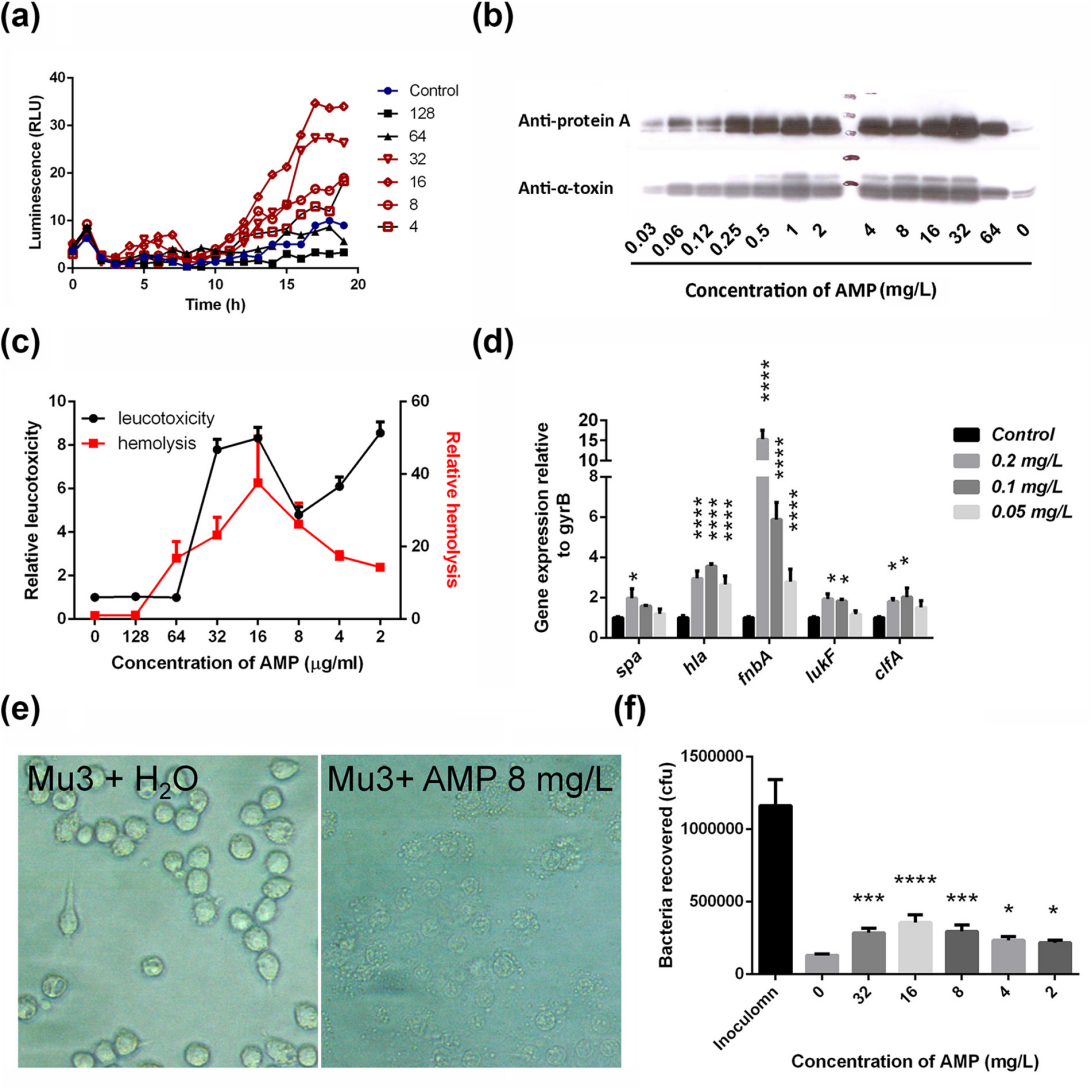
Subinhibitory concentrations of ampicillin induce the production of virulence factors *in vitro* in S. aureus Mu3. (a) Strain Mu3 harboring pGLhla was treated with various concentrations (mg/L) of ampicillin. Luminescence signals were monitored every 1 h, and the curve was plotted for *hla* gene expression. (b) Western blot analysis of S. aureus culture supernatant showing protein A and alpha-toxin production. A dose dependent increase in the production of these virulence factors was observed until 32 mg/L of ampicillin. (c) Leukotoxicity (macrophage J774.1 cells) and hemolytic (human red blood cell) activity of Mu3 culture supernatants grown at different concentrations of ampicillin. (d) qPCR analysis of virulence genes expression at subinhibitory concentrations of ampicillin. (e), Microscopic analysis of J774.1 macrophage cells treated with bacterial culture and observed under the microscope (400×). Culture supernatants of bacteria treated with 8 mg/L ampicillin or water control. (f) Intracellular bacteria recovered from ampicillin treated J774.1 macrophages. After bacteria internalized in macrophage J774.1, different concentrations of ampicillin were applied for 22h and intracellular S. aureus survival were measured by viable count. One-way ANOVA was used to analyze the luminescence signals on agar plates by multiple comparisons of different groups with control group. Data represent mean values ± SD (*, *P* < 0.05; **, *P* < 0.01; ***, *P* < 0.001; ****, *P* < 0.0001). AMP: ampicillin.

Since ampicillin is still in the guideline for empirical treatment for Methicillin susceptible S. aureus (MSSA) (12), the enhancement of virulence gene expression by antibiotics *in vitro* was first tested using ampicillin. Increased expression of virulence in the presence of ampicillin was assessed using luminescence reporter assay, q-PCR, Western blot, hemolysis, leucotoxic and intracellular survival assays. The MIC of ampicillin against S. aureus Mu3 (MRSA strain) was 64 mg/L and it started to induce *hla* expression from 32 mg/L (Fig. 1a). Based on Western blot analysis, we noticed that ampicillin dramatically enhanced protein A and alpha-toxin production in Mu3 at concentrations ranging from 0.06 mg/L to 64 mg/L (Fig. 1b). It is well known that hemolysins produced by S. aureus cause hemolysis of erythrocytes. Hence, we tested the hemolytic activity of culture supernatant grown in the presence of a subinhibitory concentration of ampicillin (16 mg/L). A 35-fold increase in hemolytic activity of human erythrocytes was observed at this concentration (Fig. 1c). Genes related to S. aureus surface protein and toxins such as *spa*, *hla*, *fnbB*, *lukF-PV* and *clfA* showed heightened expression at subinhibitory concentration of ampicillin (Fig. 1d). Leucotoxicity assay was performed using Mu3 culture on J774.1 macrophage cells. Compared to the PBS-treated control, ampicillin (32 mg/L) treated samples showed 8-fold increase in leukotoxicity and this observation validated the induction of virulence by ampicillin (Fig. 1c). Microscopic examination of macrophages (Fig. 1e) indicated that bacterial cultures treated with ampicillin at subinhibitory concentrations markedly enhanced the lysis of macrophages.

The intracellular survival of S. aureus in macrophages is dependent on multiple virulence factors such as alpha-toxin, adhesins, and aureolysin (13). Increased expression of alpha-toxin and adhesins (mainly protein A, ClfA and FnbA) (Fig. 1d) at subinhibitory concentrations of ampicillin led to the hypothesis that this antibiotic may induce the intracellular survival of S. aureus in macrophages. In the absence of ampicillin, after 24h of incubation, 90% of intracellular S. aureus were cleared by macrophages. In contrast, after infection with S. aureus, ampicillin treatment at concentrations ranging from 2 to 32 mg/L increased intracellular bacterial survival by 2- to 3-fold (Fig. 1f). This observation indicates that ampicillin at subinhibitory concentrations may impair the clearance of S. aureus in macrophages, and we anticipated that similar effects could also be observed *in vivo*. Hence, the aggravation of S. aureus virulence in the presence of ampicillin and other antibiotics was further tested in mice infection models.

### Exacerbated S. aureus infection in mice is not associated with the side effects of ampicillin.

**FIG 2:**
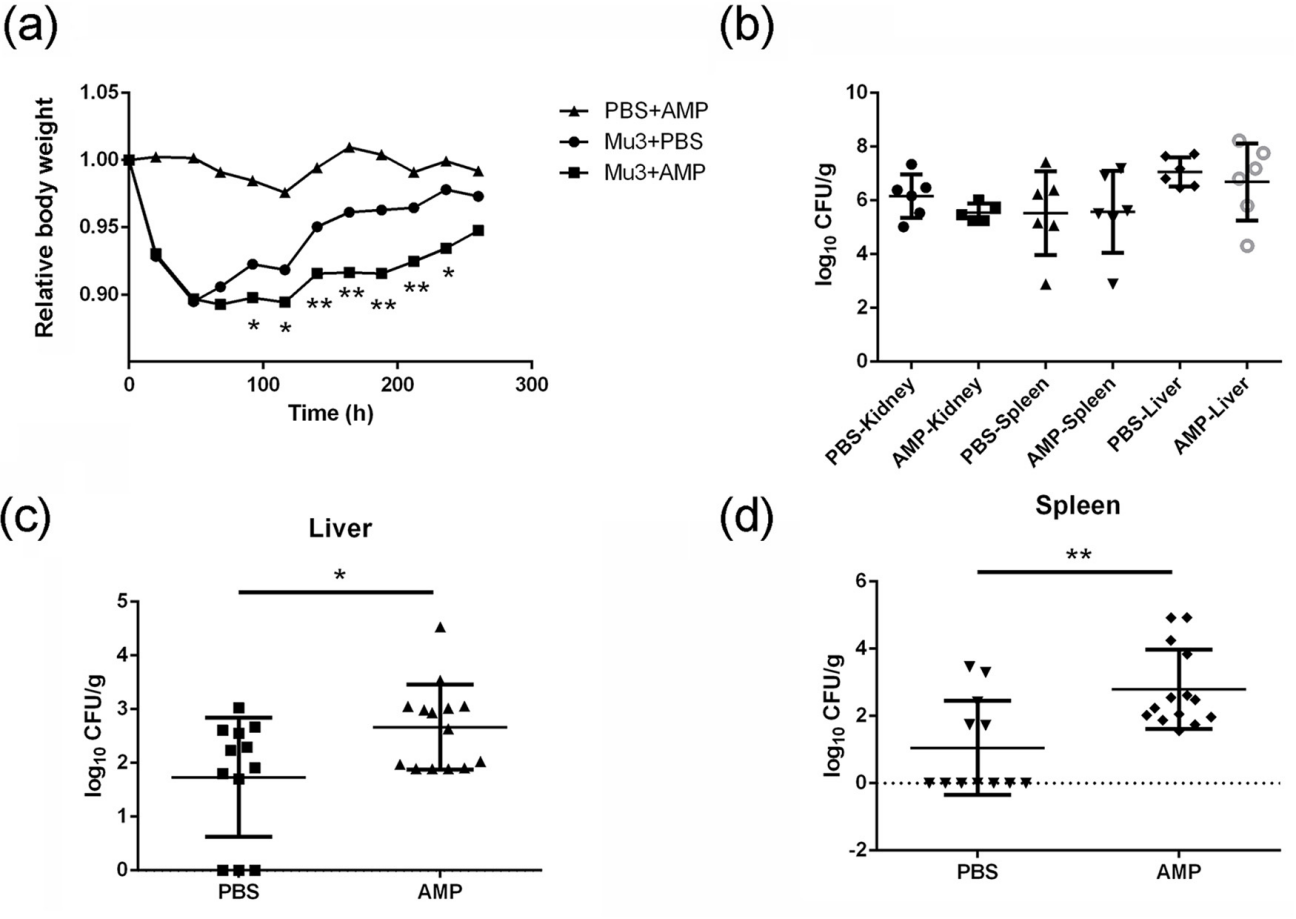
Subinhibitory concentrations of ampicillin induce virulence *in vivo* in S. aureus Mu3. In a peritonitis infection model, mice were treated with ampicillin or PBS, and bodyweight was monitored for 12 days. Livers and spleens were collected on day 6 for viable count. (a) Body weight of mice in a peritonitis infection model with various treatments. (b) Bacterial load from peritonitis model on day 3. After intraperitoneal infection, on day 3, from vehicle and ampicillin treatment groups, bacterial load was determined from different organs. (c and d) Bacteria recovered from, liver (c) and spleen (d) on day 6. Data represent mean values ± SD (*, *P* < 0.05; **, *P* < 0.01) and Student's *t* test was used for determining the statistical significance.

Studies have shown that after the administration of one dosage of ampicillin (40 mg/kg) subcutaneously, its highest serum concentration was around 30 mg/L in the mice and serum concentration retained higher than 3 mg/L for more than 2 h (14). In our Western blot analysis, we found that 0.06 mg/L of ampicillin was sufficient to induce virulence. Hence, to attain a clinically relevant concentration (15) ampicillin at 40 mg/kg/dose was applied subcutaneously in mice and bacteremia was established by infecting with S. aureus. On day 3, in contrast to the treatment group, mice from the control group showed early recovery. From day 4 to day 10, a significant difference in the loss of body weight was observed between the two groups (Fig. 2a). However, the noninfected mice, treated with same amount of ampicillin alone, did not lose body weight. Thus, ampicillin treatment at subinhibitory dosages (comparable to clinical dosage) remarkably prolonged the period of convalescence. This confirmed that the worsened outcome in mice is not associated with the side effects of ampicillin treatment (Fig. 2a).

With respect to bacterial load in kidneys, liver and spleen, on day 3, no significant differences were observed between the two groups. (Fig. 2b). However, on day 6, in the ampicillin treated group, more bacteria were recovered from the liver (*P* = 0.0156) and spleen (*P* = 0.0021) (Fig. 2c and d). There were a 1-log and 2-log bacterial load differences in the liver and spleen, respectively. The increased bacterial load further confirmed that the ampicillin treatment at subinhibitory concentrations may enhance S. aureus virulence and pathogenesis in mice. These data indicate that the worsened outcomes of infection were solely caused by the introduction of ampicillin, but not due to its side effects.

### Antibiotics induced virulence in Mu3 and clinical strains.

**FIG 3:**
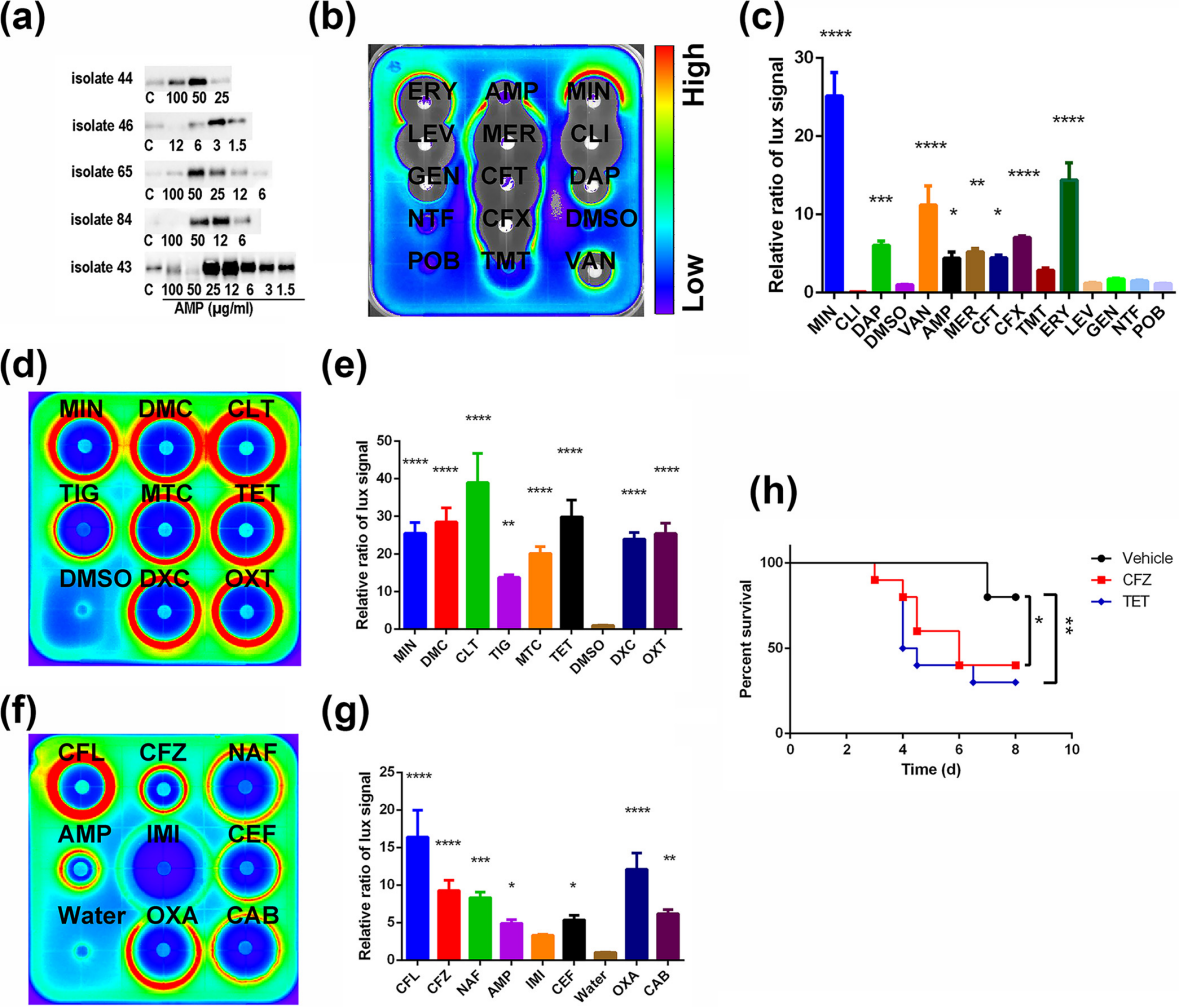
β-lactam antibiotics and tetracyclines induce the expression of virulence factors in MRSA *in vitro* and worse the infection *in vivo*. (a) Western blot showing protein A production in clinical MRSA isolates treated with different concentrations of ampicillin. (b and c) Using disc-diffusion based luminescence assay, different classes of antibiotics were tested for *hla* promoter activity in clinical isolate MRSA 34. The quantified luminescence signal of paper disc region (c). (d and e) Eight antibiotics from each class of tetracyclines were analyzed for their effects on *hla* promoter activity by disc diffusion assay in clinical isolate 34. The quantified luminescence signal of paper disc region (e). (f and g) Eight antibiotics from each class of β-lactam antibiotics were analyzed for their effects on *hla* promoter activity by disc diffusion assay in clinical isolate 34. The quantified luminescence signal of paper disc region (g). (h) Tetracycline (8 mg/kg/dose) and ceftazidime (33 mg/kg/dose) were evaluated in a bacteremia model infected with Mu3 in BALB/c mice. The survival of mice was monitored for 7 days, and data were analyzed by survival analysis. One-way ANOVA was used to analyze the luminescence signals on agar plates by multiple comparisons of different groups with control group. (*, *P* < 0.05; **, *P* < 0.01; ***, *P* < 0.001; ****, *P* < 0.0001). ERY: erythromycin; LEV: levofloxacin; GEN: gentamicin; NTF: nitrofurazone; POB: polymyxin B; MER: meropenem; CFT: cefotaxime; CFX: cefoxitin; TMT: trimethoprim; MIN: minocycline; CLI: clindamycin; DAP: daptomycin; VAN: vancomycin; TIG: tigecycline; DMC: demeclocycline; MTC: methacycline; DXC: doxycycline; CLT: chlortetracycline; TET: tetracycline; OXT: oxytetracycline; CFL: cefaclor; CFZ: ceftazidime; IMI: imipenem; OXA: oxacillin; NAF: nafcillin; CEF: ceftriaxone; CAB: carbenicillin.

The enhanced S. aureus virulence caused by ampicillin prompted us to test similar effects in other strains with different classes of antibiotics. Using a panel of clinical isolates, we clearly observed the production of protein A induced by different concentrations of ampicillin (Fig. 3a, Fig. S1). Later, in these isolates, we tested whether other classes of antibiotics will also show similar induction effect. Interestingly, along with β-lactam antibiotics, tetracyclines also induced virulence in most of the clinical isolates (Fig. 3b and c, Table 1). Using antibiotics belonging to these two classes, we identified all selected antibiotics heightening the activity of *hla* promoter, (Fig. 3d to g, Table 1) which plays a pivotal role in virulence. In bacteremia model, similar to ampicillin, tested antibiotics in these two classes at clinical dosage, worsened the infection (Fig. 3h, Fig. S2 a-h). However, when these antibiotics at same doses were administered on uninfected mice, they did not affect the body weight (Fig. S2i). Thus, our findings suggested that the tested antibiotics had a virulence induction effect, and clinical S. aureus isolates may have comparable responses to these antibiotics.

**TABLE 1:** Inhibition zone and modulating effects of different antibiotics against different clinical isolates^*a*^

Inhibition zone to different strains (mm)																												
Abbr.	Generic name	Class	Isolate 14	Isolate 15	Isolate 22	Isolate 24	Isolate 25	Isolate 34	Isolate 42	Isolate 43	Isolate 44	Isolate 45	Isolate 46	Isolate 63	Isolate 64	Isolate 65	Isolate 66	Isolate 72	Isolate 73	Isolate 76	Isolate 83	Isolate 84	Isolate 85	Isolate 86	Isolate 509	Isolate 513	Mu3	USA300
ERY	Erythromycin	Macrolides(Bs)	22	26	8	22	/	23	7	24	25	7	25	22	25	27	22	24	/	25	22	25	25	24	22	10	/	13
LEV	Levofloxacin	Quinolones	25	29	15	15	16	16	9	18	26	8	28	22	28	29	22	27	24	27	25	28	26	25	30	14	15	16
GEN	Gentamicin	Aminoglycosides	16	19	/	17	7	18	17	17	18	8	18	17	20	21	17	19	18	18	16	18	19	19	18	/		20
NTF	Nitrofurazone	Nitrofurans	7	7	8	7	8	7	8	8	7	7	7	7	7	9	/	9	7	9	7	7	/	7	/	/	/	/
POB	Polymyxin B	Polypeptides	7	7	7	7	/	7	7	/	/	/	/	8	7	7	/	7	7	7	/	7	7	7	7	/	/	/
AMP	Ampicillin	Penicillins	/	7	/	/	/	/	/	16	17	/	16	23	/	14	/	/	/	/	/	/	9	12	/	/	/	8
MER	Meropenem	Carbapenems	25	28	25	22	24	24	26	31	24	24	33	32	35	42	31	32	33	32	35	34	30	34	/	/	/	/
CFT	Cefotaxime	Cephalosporins	20	27	19	14	17	16	18	26	32	17	30	28	31	31	27	30	27	28	30	28	23	28	12	/	/	14
CFX	Cefoxitin	Cephalosporins	17	17	15	15	14	17	17	21	27	15	26	25	24	24	22	25	24	23	26	25	26	27	13	/		15
TMT	Trimethoprim	Sulfonamides	23	22	24	20	/	21	17	20	20	22	22	18	18	22	15	22	24	19	22	17	23	21	14	/	/	13
MIN	Minocycline	Tetracyclines	24	24	22	22	22	21	23	22	24	23	22	23	26	29	22	23	24	22	28	24	25	24	22	17	8	22
CLI	Clindamycin	Lincosamides	24	30	28	16	/	27	28	27	30	28	27	28	29	33	24	25	26	23	18	28	30	30	26	28	/	/
DAP	Daptomycin	Lincosamides	16	18	18	17	16	18	18	16	16	19	17	17	17	20	16	18	16	17	15	16	18	18	15	17	17	17
VAN	Vancomycin	Glycopeptides	15	16	17	15	15	16	16	15	15	16	15	15	16	17	15	17	15	16	15	15	17	16	15	16	17	16
																												
NAF	Nafcillin	β-lactam	16	30	12	12	18	14	11	28	32	13	30	25	N	20	24	20	22	24	25	25	19	N	21	/	20	15
CEF	Ceftriaxone	β-lactam	11	24	15	13	19	15	13	27	25	18	25	24	N	24	22	20	22	17	22	25	25	N	14	/	/	17
CAB	Carbenicillin	β-lactam	/	10	/	/	/	/	/	27	23	/	22	27	N	10	/	/	8	8	8	11	10	N	/	/	22	16
CFZ	Ceftazidime	β-lactam	10	22	13	10	12	10	10	19	23	12	20	16	N	17	16	12	16	19	15	18	12	N	15	/	/	15
IMI	Imipenem	β-lactam	23	35	28	24	27	23	27	28	44	40	42	45	N	45	35	27	30	29	40	32	24	N	32	/	28	26
OXA	Oxacillin	β-lactam	10	23	10	8	17	8	10	30	32	10	28	25	N	28	18	16	20	23	17	26	20	N	/	/	23	14
CFL	Cefaclor	β-lactam	/	15	/	/	/	/	/	24	24	/	25	24	N	15	10	/	12	11	10	15	13	N	/	/	/	11
AMP	Ampicillin	β-lactam	/	/	/	/	/	/	/	15	16	/	11	15	N	/	/	/	/	/	/	/	/	N	/	/	/	13
																												
CLT	Chlortetracycline	Tetracyclines	28	34	11	31	9	12	29	29	9	27	28	29	29	11	11	29	9	25	26	28	11	29	20	/	7	11
TET	Tetracycline	Tetracyclines	29	34	8	31	8	10	28	28	8	26	29	28	29	8	8	29	8	29	26	28	9	30	20	/	/	7
OXT	Oxytetracycline	Tetracyclines	29	36	/	31	7	/	27	27	/	28	27	27	28	7	/	29	8	25	25	28	8	28	18	/	/	7
DMC	Demeclocycline	Tetracyclines	28	35	9	32	9	12	29	29	9	28	29	29	29	9	9	28	8	27	25	29	10	28	20	/	7	8
MTC	Methacycline	Tetracyclines	29	36	9	32	9	12	28	28	8	28	28	28	30	10	10	30	9	28	27	29	11	31	20	/	8	8
DXC	Doxycycline	Tetracyclines	30	37	22	32	16	18	30	30	17	24	30	30	29	14	14	31	17	28	26	29	16	30	20	7	13	14
MIN	Minocycline	Tetracyclines	27	30	30	29	28	26	26	27	28	27	26	26	26	27	24	27	27	26	23	27	27	28	18	18	10	24
TIG	Tigecycline	Tetracyclines	25	27	28	29	23	31	22	23	23	22	22	23	24	21	20	23	23	22	23	24	24	22	17	17	17	21
Induction or repression against different strains (1 to 9: induction; −9 to −1: repression)																												
abbr.	Generic name	Class	Isolate 14	Isolate 15	Isolate 22	Isolate 24	Isolate 25	Isolate 34	Isolate 42	Isolate 43	Isolate 44	Isolate 45	Isolate 46	Isolate 63	Isolate 64	Isolate 65	Isolate 66	Isolate 72	Isolate 73	Isolate 76	Isolate 83	Isolate 84	Isolate 85	Isolate 86	Isolate 509	Isolate 513	Mu3	USA300
ERY	Erythromycin	Macrolides(Bs)	1	4	−1	−5	0	−3/2	1	6	5	1	2	−5	2	−5	−5	1	0	1	−5	−5	−5	−5	3	0	0	−5
LEV	Levofloxacin	Quinolones	3	4	1	1	1	2	3	3	1	1	2	1	3	−4	−3	2	−3	−2	2	−3	−3	−4	5	5	4	5
GEN	Gentamicin	Aminoglycosides	1	3	0	−3	0	1	1	1	1	1	1	−1	−3	−3	−2	1	1	0	−2	−3	−2	−3	2	0	0	3
NTF	Nitrofurazone	Nitrofurans	2	1	0	1	1	1	0	0	0	0	1	0	4	1	0	2	3	3	0	0	2	0	0	0	0	1
POB	Polymyxin B	Polypeptides	1	0	0	0	0	0	0	0	0	0	0	0	2	0	0	0	1	−2	0	0	0	0	0	5	4	1
AMP	Ampicillin	Penicillins	2	1	1	1	1	2	1	3	2	3	2	9	2	2	2	2	2	2	2	3	3	2	0	0	4	8
MER	Meropenem	Carbapenems	5	4	5	7	5	5	5	6	5	6	6	6	7	7	7	9	9	9	9	3	3	4	5	2	3	6
CFT	Cefotaxime	Cephalosporins	6	4	4	7	5	5	4	5	4	5	5	5	6	7	5	8	9	8	7	5	5	5	7	3	3	4
CFX	Cefoxitin	Cephalosporins	8	5	6	9	5	7	7	6	4	7	5	6	5	9	6	9	9	9	8	6	1	6	8	4	8	7
TMT	Trimethoprim	Sulfonamides	2	5	1	3	0	2	6	3	3	3	2	−1	6	1	0	5	−2	4	2	−3	−2	−4	4	3	3	4
MIN	Minocycline	Tetracyclines	5	7	−5	4	3	5	6	8	8	4	9	6	6	1	6	6	7	6	1	−5	−2	−5	6	4	4	6
CLI	Clindamycin	Lincosamides	−5	−5	−7	−7	0	−7	−8	−5	−5	−7	−5	−7	−6	−5	2	2	−7	0	5	−7	−7	−7	−5	−8	0	0
DAP	Daptomycin	Lincosamides	2	4	1	4	3	4	4	5	4	0	4	1	2	4	2	6	2	4	2	1	2	1	3	6	5	6
VAN	Vancomycin	Glycopeptides	2	4	3	6	4	5	5	6	4	4	4	2	2	4	3	5	4	5	3	3	4	3	3	8	6	6
																												
NAF	Nafcillin	β-lactam	9	8	9	9	8	9	9	6	6	7	6	7	N	9	N	9	9	9	9	9	7	8	9	9	6	6
CEF	Ceftriaxone	β-lactam	7	3	7	4	3	3	3	6	6	4	6	5	N	6	N	8	8	8	6	7	7	7	5	4	3	4
CAB	Carbenicillin	β-lactam	0	3	2	1	1	2	0	6	4	0	5	7	N	2	N	1	0	0	0	0	2	1	0	0	7	6
CFZ	Ceftazidime	β-lactam	5	5	7	6	5	7	6	6	6	5	6	5	N	4	N	6	7	7	5	6	4	6	4	5	5	4
IMI	Imipenem	β-lactam	5	0	6	8	0	7	6	4	4	3	3	3	N	3	N	4	3	5	5	5	5	0	7	4	3	4
OXA	Oxacillin	β-lactam	7	5	8	8	5	8	7	8	7	5	8	7	N	5	N	6	4	7	5	4	5	7	3	5	5	5
CFL	Cefaclor	β-lactam	3	5	1	1	4	5	2	9	9	2	9	9	N	8	N	5	3	5	0	3	4	3	0	5	5	7
AMP	Ampicillin	β-lactam	1	1	1	1	2	1	0	7	4	1	5	8	N	1	N	1	1	0	0	0	2	0	0	2	4	7
																												
CLT	Chlortetracycline	Tetracyclines	6	6	3	6	8	7	6	7	8	5	7	8	6	7	5	5	8	6	5	5	5	5	5	0	6	6
TET	Tetracycline	Tetracyclines	6	6	2	4	6	3	3	7	7	0	7	7	6	5	3	3	8	4	5	5	4	5	5	0	1	2
OXT	Oxytetracycline	Tetracyclines	6	6	0	3	6	2	0	7	7	0	8	5	6	5	0	0	8	1	5	4	3	5	4	0	6	3
DMC	Demeclocycline	Tetracyclines	6	6	3	5	7	5	5	7	8	4	7	7	6	5	4	4	8	6	5	5	5	4	5	0	4	1
MTC	Methacycline	Tetracyclines	5	4	2	4	5	4	4	6	6	0	5	5	6	3	3	4	6	5	5	4	4	3	4	−4	3	1
DXC	Doxycycline	Tetracyclines	6	6	3	7	6	6	7	7	7	5	7	6	6	5	6	5	8	7	4	6	6	5	4	0	6	4
MIN	Minocycline	Tetracyclines	6	5	0	9	8	9	6	7	8	8	6	8	5	5	7	7	7	8	5	4	5	4	4	6	3	3
TIG	Tigecycline	Tetracyclines	3	4	0	9	5	8	4	4	5	7	6	5	4	5	5	6	5	6	2	3	6	2	4	6	2	3

a / means no inhibition.

### Ceftazidime, ampicillin, and tetracycline induced virulence *in vivo*.

**FIG 4:**
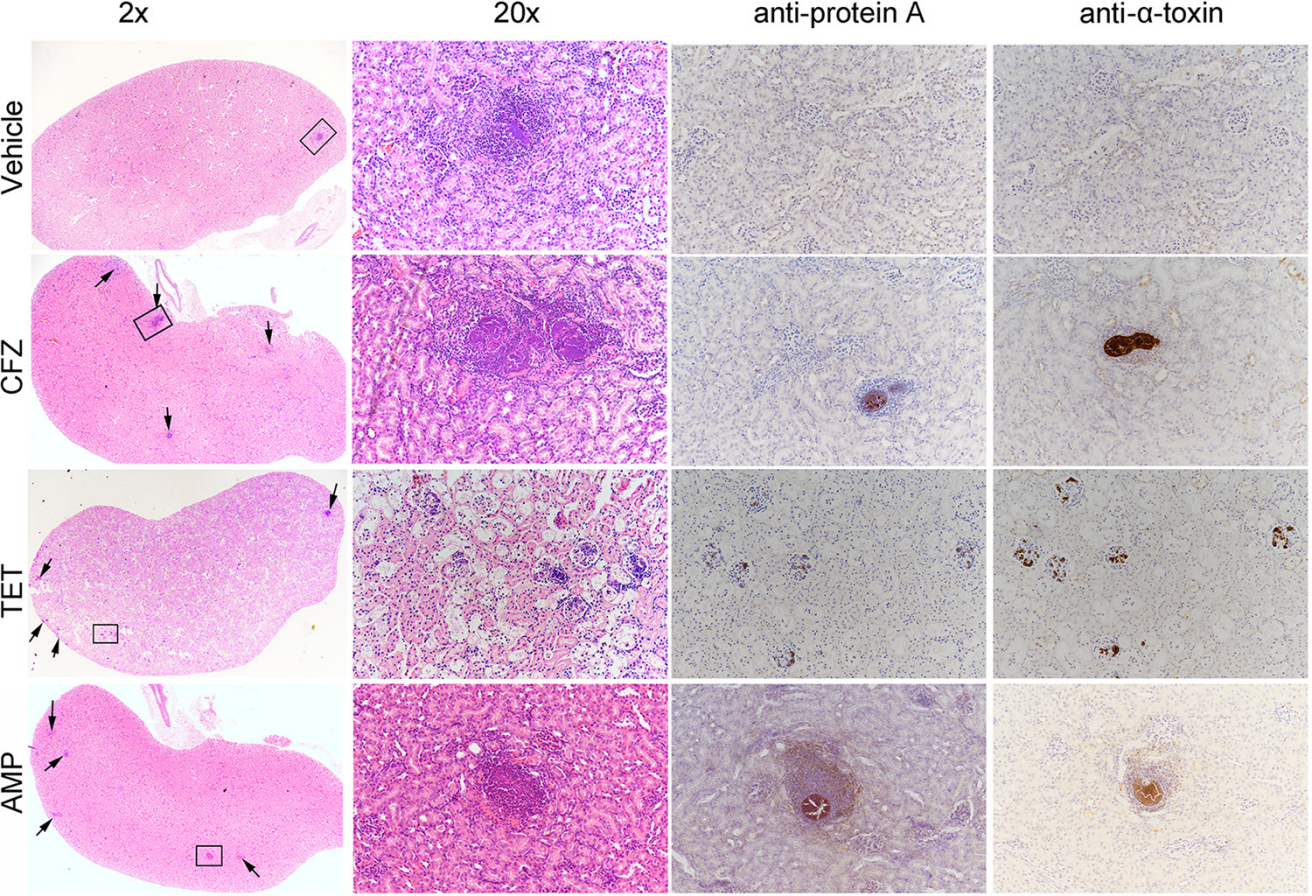
Histological analysis of antibiotic induced virulence in kidneys. Histological examination revealed the formation of abscess in ceftazidime, ampicillin and tetracycline treated groups. Photos with ×2 magnification shows the gross area of the examined tissue and abscesses are indicated by arrows. The 20× magnified photos represent the selected abscess and the staining of cells at the abscess site can be clearly seen. The production of protein A and alpha-toxin was measured by immunohistochemistry and this is visible in the same abscess region.

Histological examination of mice kidneys infected with S. aureus and treated with ampicillin and ceftazidime revealed more abscesses than those for vehicle group (ceftazidime: 15 ± 4.2; Ampicillin: 9.6 ± 4.9; Control: 4.2 ± 1.8). Whereas tetracycline treated mice displayed small and numerous abscesses (54.6 ± 21.2) (Fig. 4). Infected mice treated with these antibiotics also showed higher production of protein A and alpha-toxin in their kidneys than those for vehicle group (Fig. 4). Although ceftazidime and tetracycline showed remarkable variation in abscess formation, their bacterial load in kidneys were identical on day 2, (Fig. S2j). This indicates that virulence in MRSA strain Mu3 is produced entirely by the treated antibiotics and is unaffected by bacterial load. These observations substantiate that when antibiotics such as ceftazidime, ampicillin and tetracycline were used at subinhibitory concentrations, not only would they fail to save the infected mice, but they may also induce S. aureus virulence leading to aggravated infection.

## DISCUSSION

In this study, we showed that antibiotics can increase S. aureus pathogenicity *in vitro* and *in vivo*. The induction of virulence factors by antibiotics in laboratory and clinical S. aureus strains was identified using a luminescence reporter system, cell invasion investigations, protein-based tests and in mouse peritonitis model and bacteremia model.

Numerous studies have shown that the antibiotic ampicillin can induce virulence in S. aureus (16, 17). Like prior studies, ampicillin at subinhibitory concentrations enhanced S. aureus *hla* expression *in vitro*. Despite this evidence, ampicillin is still used alone or in combination with macrolides or tetracycline during empirical treatment (12, 18). In some clinical settings, MRSA patients are treated with vancomycin and β-lactam antibiotics (19–21). Since ampicillin reaches the infection site earlier than vancomycin (22, 23), the bacterial virulence would already have been induced by ampicillin before vancomycin could kill the bacteria. Due to this potent virulence induction activity of ampicillin, we anticipated that β-lactam antibiotics may adversely affect clinical outcomes. We found that ampicillin, ceftazidime, and tetracycline increased S. aureus virulence genes expression and their production *in vitro*, and increased pathogenesis *in vivo*.

The induction of virulence by antibiotics was tested using multiple strains. Off note, we specifically used MRSA strain Mu3 for testing the virulence expression *in vivo*. Compared to other MRSA strains, the basic expression level of alpha-toxin in Mu3 is lower. Meanwhile, the concentration window for the response to ampicillin is broad ranging from 0.06 μg/mL to 32 μg/mL. These properties allowed us to demonstrate observable virulence induction effect *in vivo* using Mu3 strain and ampicillin.

Since ampicillin is not the strongest antibiotic in term of induction of *hla* expression as observed by disc diffusion assay, we wanted to test other antibiotics for virulence induction under *in vitro* and *in vivo* conditions. Antibiotics which show stronger induction effect on virulence expression *in vitro* may substantially worsen the infection *in vivo*. Hence, ceftazidime and tetracycline were assessed for virulence induction *in vivo*. It is noteworthy to mention that the antibiotics ceftazidime and tetracycline aggravated the disease outcome during the therapy, indicating that virulence induction is common for different antibiotics. Although the majority of the tested strains showed antibiotic-induced virulence, some did not respond to subinhibitory concentrations of ampicillin. Thus, the antibiotic-induced virulence is strain dependent.

Another limitation of this study is the use of mouse models. Mice and rats are widely used to assess antibiotic effectiveness or to investigate S. aureus pathogenesis *in vivo*. However, the mouse neutrophil is resistant to many S. aureus toxins, such as PVL. Hence, mouse may not be the optimal model for studying staphylococcal pathogenesis. Apart from mouse, the rabbit is an optimum model for studying S. aureus pathogenesis. Compared to human cells, rabbit cells are more susceptible to S. aureus toxins. For example, rabbit erythrocytes and neutrophils are more susceptible to alpha-toxin and PVL, respectively (24, 25). Some infection models such as osteomyelitis, employ larger animals like dogs, ovine, goats, and pigs. Despite the advantages of using rabbits and larger animals, we could not use these animals in our study due to their body size and special requirements by animal facility. Additionally, the need for higher sample size of these animals for infection studies may pose more challenges. Replicating these experiments in rabbits or in clinical settings may result in even worse outcomes because several generated S. aureus toxins cannot be represented in mice models. Despite these limitations, we believe that our investigation has provided an insight about the complications associated with antibiotics and may direct us to use nonantibiotic therapies against pathogenic bacteria.

Peritonitis and bacteremia are two life-threatening infections caused by S. aureus. Our mice peritonitis and bacteremia models have successfully illustrated that S. aureus infections were severely exacerbated after treatment with selected antibiotics at subinhibitory concentrations. Apart from the three antibiotics (AMP, CFZ, and TET) examined in our animal studies, there were other antibiotics that exhibited higher virulence-inducing properties in the paper disc experiment (Fig. 3), suggesting that similar or even worse outcomes may occur in real clinical scenarios. In clinical practice, clinicians are required to provide treatment plans prior to the identification of the pathogens or the availability of the antibiogram, which means that patients may receive erroneous antibiotics or subinhibitory concentrations of antibiotics. Even though the antibiotics that we tested are not first line antibiotics for confirmed MRSA cases, these antibiotics are nonetheless included in the guideline for empirical therapy (12, 18).

We believe that additional research on the antibiotic-induced virulence in combinatorial therapy is essential. For example, S. aureus pathogenesis during combinatorial treatment with β-lactam and other antibiotics could be evaluated in animal models. Antibiotics could be chosen based on local epidemiology and national guidelines and except in severe necrotic cases, combined therapy is rarely necessary. There may be a theoretical rationale for combining two or three antibiotics in severe infections with signs of toxic shock, necrotizing fasciitis, or purpura fulminans (26). When antibiotics are used in combination, subinhibitory concentrations are feasible, particularly for some deep infections. However, this may lead to virulence induction and emergence of multidrug-resistant isolates (27). Due to the broad-spectrum activity and relatively less side effects, β-lactam antibiotics are widely used in the antimicrobial therapy. Since clindamycin at subinhibitory concentrations displays anti-virulence property (16), combination of β-lactam antibiotics with clindamycin has been widely used in antimicrobial therapy. But S. aureus rapidly develops inducible clindamycin resistance, particularly in CA-MRSA, limiting the utility of clindamycin as an empirical treatment (28, 29). In these strains, clindamycin may fail to display anti-virulence property. Off note, in our study clindamycin did not show any anti-virulence effect in clindamycin resistant clinical isolates (Table 1).

Collectively, our findings provided compelling evidence that the worsened infections resulted from induced staphylococcal virulence by subinhibitory antibiotic doses. Our *in vivo* findings comprehended that antibiotic-induced S. aureus infections are extremely serious and judicious use of antibiotics is essential. Based on our findings, we emphasize on the restricted use of antibiotics not only for empirical treatment but also for clinical and retrospective studies. Apart from animal experiments, population-based epidemiological studies are needed to explore the marked influence of antibiotics in clinical outcomes of patients during empirical treatment.

## MATERIALS AND METHODS

### Bacterial strains and plasmids.

The bacterial strains used in this study are listed in Table 2. Brain heart infusion (BHI) broth and BHI agar plates were used throughout to grow S. aureus. Chloramphenicol was used at 10 μg/mL. Unless otherwise stated, all cultures were grown aerobically at 37°C with shaking, and growth was monitored at 600 nm with a HITACHI U-2800 (Hitachi, Japan) spectrophotometer.

**TABLE 2:** Strains and plasmids used in this study

Strain	Phenotype	Spa typing	Source
Lab strains			
RN6390	MSSA, Agr−		Lab stock
Newman	MSSA, Agr+		Lab stock
COL	MRSA, Agr−		Lab stock
USA300 FPR 3757	CA-MRSA, Agr+		ATCC ABB1776
Mu3	MRSA, Agr+		ATCC700698
Clinical isolates			
AE052	CA-MRSA, Agr+		This study
ST45	MRSA, Agr+		This study
ST239A	MRSA, Agr+, isolate 509		This study
ST239AH	MRSA, Agr+, isolate 513		This study
Isolate 14	Clinical isolate	T1170	This study
Isolate 15	Clinical isolate	T1081	
Isolate 22	Clinical isolate	T1081	This study
Isolate 24	Clinical isolate	T1081	This study
Isolate 25	Clinical isolate	T062	This study
Isolate 34	Clinical isolate	T1081	This study
Isolate 42	Clinical isolate	T1081	This study
Isolate 43	Clinical isolate	T211	This study
Isolate 44	Clinical isolate	T211	This study
Isolate 45	Clinical isolate	T1081	This study
Isolate 46	Clinical isolate	T211	This study
Isolate 63	Clinical isolate	T211	This study
Isolate 64	Clinical isolate	T065	This study
Isolate 65	Clinical isolate	T4398	This study
Isolate 66	Clinical isolate	T091	This study
Isolate 72	Clinical isolate	T091	This study
Isolate 73	Clinical isolate	T034	This study
Isolate 83	Clinical isolate	T548	This study
Isolate 84	Clinical isolate	T127	This study
Isolate 85	Clinical isolate	T189	This study
Isolate 86	Clinical isolate	T002	This study
Plasmid			
pGL	*gfp-luxABCDE* dual reporter plasmid		Lab stock
pGL*hla*	*gfp-luxABCDE* dual reporter driven by *hla* promoter		Lab stock
pGL*spa*	*gfp-luxABCDE* dual reporter driven by *spa* promoter		Lab stock

### MIC tests.

MIC was determined by inoculating 5 × 10^4^ S. aureus cells in 100 μL BHI medium on 96-well plates with a serial dilution of antibiotics. The MIC was defined as the minimum concentration resulting in a cell density less than 0.01 OD at 620 nm (16, 30), which corresponded to no visible growth, after incubating for 18 h at 37°C.

### Measurement of gene expression by bacterial cultures.

Using the published protocol, different bacterial strains transformed with plasmid pGL*hla* or pGL*spa* (Table 2) were subjected to bioluminescence assay (7). In brief, 100 μL (10^6^ CFU/mL) of S. aureus samples in triplicate were dispensed into clear-bottom 96-well microtiter plates and incubated at 37°C. The bacterial growth was assessed by measuring the optical density at 620 nm (OD_620_). For bioluminescence, lux reading was taken every 30 min using DTX 800/880 multimode plate reader (Beckman).

### Disk diffusion and lux assays.

A single colony of bioluminescent S. aureus from BHI agar was resuspended in 200 μL of sterile water. Immediately, this suspension was added to 75 mL of 0.7% (wt/vol) soft agar (375-fold dilution of original suspension), mixed thoroughly and overlaid onto BHI agar plates. Five μL of antibiotics at 4 mM concentration were added to each paper disc and placed on the plates overlaid with bacterial soft agar. The plates were incubated at 37°C, and after 20 h, inhibition zones were measured, and luminescence was detected with a PE IVIS Spectrum *in vivo* imaging system (PerkinElmer).

### Real-time PCR to verify expression levels.

Using RNeasy kit (Qiagen, Germany), and by following the manufacturer's protocol, RNA from S. aureus strains was extracted (7). Contaminating chromosomal DNA was removed by DNase treatment (Life Technologies, Hong Kong). Purified S. aureus RNA was reverse transcribed into cDNA by PrimeScript RT reagent kit (TaKaRa, Japan) and then subjected to real‐time PCR analysis using a Vii7 thermocycler (life technologies, Hong Kong) and SYBR Premix Ex taq (TaKaRa, Japan). A relative quantification of S. aureus transcripts was determined by measuring the ratio of expression of target transcripts to expression of *gyrB* (housekeeping or calibration gene). The sequence of primers used in real-time PCR experiments are mentioned in Table 3.

**TABLE 3:** Primers used in this study

Gene	Primer for Real-time PCR
rt-*hla*-f	AAAAAACTGCTAGTTATTAGAACGAAAGG
rt-*hla*-r	GGCCAGGCTAAACCACTTTTG
rt-*spa*-f	CAGCAAACCATGCAGATGCTA
rt-*spa*-r	GCTAATGATAATCCACCAAATACAGTTG
rt-*fnbA*-f	ACAAGTTGAAGTGGCACAGCC
rt-*fnbA*-r	CCGCTACATCTGCTGATCTTGTC
rt-*clfA*-f	ATGTGACAGTTGGTATTGACTCTGG
rt-*clfA*-r	TAGGCACTGAAAAACCATAATTCAGT
rt-*lukF*-f	TTTAAGCTTATGAAGAGTTTCAAGTTCA
rt-*lukF*-r	CCCAACCATTAGCCATAATTTTATGT
rt-*gyrB*-f	CAAATGATCACAGCTTTGGTACAG
rt-*gyrB*-r	CGGCATCAGTCATAATGACGAT

### Western blot.

S. aureus strains were cultured in BHI broth and supernatant was collected at different time intervals. For ampicillin treated samples, after 24h of incubation, the OD600 of the culture was adjusted to 6 and the supernatant was collected by centrifugation. The collected supernatant was subjected to boiling in loading buffer. After this step, 5 μL of the culture supernatant was loaded onto a 12% sodium dodecyl sulfate-polyacrylamide gel. The Western blot protocol was performed as described in the product guide of Amersham ECL Western blotting detection reagents (GE Healthcare, Buckinghamshire, United Kingdom). Alpha-hemolysin was detected by using rabbit anti-staphylococcal α-hemolysin antibody (1:20,000) (Sigma-Aldrich) and goat Horseradish Peroxidase (HRP)-conjugated anti-rabbit IgG (1:5,000) (Sigma-Aldrich). Protein A was visualized with HRP-conjugated Rabbit anti-staphylococcal Spa antibody (1:20,000) (Abcam).

### Leucotoxic assay.

Leukotoxic assay was performed as previously described (31). In brief, J774.1 mouse macrophage cells (ATCC TIB-67) were seeded in 96-well plates with a density of 5.0 × 10^4^ cells per well. Staphylococcal culture supernatant (grown in presence or absence of antibiotics) was diluted to 10 times in DMEM and 100 μL**/**well of this mixture was added in triplicate to the cultured J774.1 cells. Following incubation at 37°C for 1 h, cell viability was measured by performing MTT (3-(4,5-dimethylthiazol-2-yl)-2,5-diphenyltetrazolium bromide) assay.

### Hemolysis assay on human red blood cells.

Isolation of human erythrocytes and hemolysis assay were performed using the published protocols (31, 32). Briefly, 50 μL of washed human erythrocytes (5 × 10^6^ cells/mL) were added to microtiter plates (Cellstar TC; Greiner, Germany). Wells were treated with 50 μL of serially diluted bacterial culture supernatant and incubated for 60 min at 37°C. ddH2O and PBS were used as positive and negative control, respectively, in each assay. Following centrifugation, the absorption at 450 nm (A450) of the resulting supernatants was determined with a Multimode detection DTX plate reader (Beckman, Germany). All experiments were performed in triplicates and three independent assays were performed to draw the conclusion.

### Intracellular survival assay.

The ability of S. aureus to persist in J774.1 mouse macrophage cells was assessed by measuring the intracellular viable count of bacteria. Briefly, prior to bacterial inoculation, wells containing J774.1 were rinsed twice with warm PBS. The overnight bacterial culture grown on BHI agar was resuspended in DMEM medium (supplemented with 1% FBS) and added to J774.1 cells with a density of ~5 × 10^6^ CFU/mL (33). After 1 h, 5 μg/mL of lysostaphin was added to remove the extracellular bacteria (34). The J774.1 cells infected with intracellular S. aureus was treated with different concentrations of ampicillin and incubated for 22 h. Following incubation, total bacteria in each well were determined by CFU analysis. Uninfected control wells which underwent the same washes were processed in parallel and served as negative controls. Wells containing medium only were used for background correction. The levels of intracellular bacterial survival in control and antibiotic treated samples were calculated by the formula: final CFU of experimental well/CFU of initial intracellular bacteria. Results were assessed by repeating the experiment three times with triplicate samples in each trial.

### Mouse peritonitis model.

As previously described (35), we kept the 6- to 8-week-old BALB/c female mice in biosafety level 2 animal facility. Mice were housed in microisolator cages, and they received food and water *ad libitum*. Standard operating procedure was followed for the ethically approved protocols (CULATR 3055-13 and 3678-15). The experiments were conducted in biosafety level 2 animal facility.

Animals were daily monitored for symptoms of disease (body weight drop, inactivity, ruffled fur and labored breath) and death. During infection, animals showing severe disease symptoms (such as loss of mobility) and loss of over 20% of body weight were euthanized by i.p. injection of 100 mg/kg pentobarbitone.

To establish peritonitis model, mid-exponential phase of S. aureus culture was washed twice with sterilized PBS and resuspended in PBS to obtain 1 × 10^8^ CFU/100 μL. Mice were i.p. injected with 4 × 10^8^ S. aureus. Six hours later, mice were randomized into two groups (*n* = 12). Each group received a dose of either 100 μL PBS or 100 μL of 8 mg/mL ampicillin in PBS subcutaneously (s.c.) twice daily (12-h interval). A third group of mice (*n* = 6) (as a control) were treated only with ampicillin without bacterial infection. To determine the postinfection viable bacterial count, 6 mice from each group were euthanized on day 3 and 12. The experiment was repeated once.

For bacterial load on day 6, the third trial of experiment was conducted and 15 mice from each group were euthanized. Kidneys, livers and spleens were harvested, homogenized in PBS, and plated on BHI agar.

### Mouse bacteremia model.

S. aureus strain Mu3 was cultured to attain the early-exponential phase, washed twice with sterilized PBS, and resuspended in PBS to obtain a cell density of 1 × 10^8^ CFU/100 μL.

The female BALB/c mice, 6 - 8-week-old, were infected through tail vein (i.v.) with S. aureus (1 × 10^8^ CFU/mouse) and randomized into 16 groups consisting of 5 mice per group. One hour after infection, mice were treated with designated concentrations of antibiotics (s.c.) or PBS (serving as control). Antibiotics used in this study are listed in Table 4 and were administered twice per day. Treatments were performed twice per day at 12-h interval. The survival was monitored according to the body condition scoring system. After selection of antibiotics, experiment was repeated twice with selected antibiotics, ampicillin (0.8 mg/dose), tetracycline (0.15 mg/dose) and ceftazidime (0.33 mg/dose).

**TABLE 4:** List of antibiotics used for animal bacteremia models

Antibiotics	Dose per mouse	Relative to human per dose	Dose (mg/kg)
Nafcillin	0.33 mg and 0.66 mg	1 g and 2 g	16 mg/kg/dose and 33 mg/kg/dose
Meropenem	0.15 mg and 0.33 mg	0.5 g and 1 g	8 mg/kg/dose and 16 mg/kg/dose
Ceftazidime	0.33 mg and 0.66 mg	1 g and 2 g	16 mg/kg/dose and 33 mg/kg/dose
Gentamicn	0.04 mg and 0.08 mg	0.125 g and 0.25 g	2 mg/kg/dose and 4 mg/kg/dose
Erythromycin	0.33 mg and 0.66 mg	1 g and 2 g	16 mg/kg/dose and 33 mg/kg/dose
Tetracycline	0.08 mg and 0.15 mg	0.25 g and 0.5 g	4 mg/kg/dose and 8 mg/kg/dose
Chloramphenical	0.15 mg and 0.33 mg	0.5 g and 1 g	8 mg/kg/dose and 16 mg/kg/dose
Vancomycin	0.15 mg	0.5 g	8 mg/kg/dose

For histological studies, experiment was repeated again, and samples were obtained from mice which had undergone survival experiments. On day 2, mice from each group were euthanized and kidneys were collected. One kidney from each mouse was fixed in formalin for histological examination. The other kidney from each mouse was homogenized in PBS and plated on BHIA to determine the bacterial viable count.

### Histology.

Kidney samples collected from i.v. lethal infection model was stored in 10% formalin for 48h and rinsed with 70% ethanol. Tissues were embedded in paraffin, thin-sectioned, stained with hematoxylin and eosin (H&E) or immunohistochemistry (IHC) and examined by microscopy (36). For IHC, antibodies which were used for Western blot analysis, were applied at different dilutions. α-Toxin was detected with rabbit anti-staphylococcal alpha-toxin antibody (1:10,000) (Sigma-Aldrich) and goat Horseradish Peroxidase (HRP)-conjugated anti-rabbit IgG (1:10,000) (Sigma-Aldrich).

### Statistics.

Statistical analysis was performed using Graph Pad Prism version 7.0. All error bars depict the standard deviation (SD). Horizontal lines depict the mean. All replicates are biological (from different samples). One-way ANOVA was used to do multiple comparisons of different groups. Student's *t* test was used to determine the statistical significance of bacterial load in animal experiment. Survival analysis was used for analyzing difference between different groups in lethal animal model.
