# Supplementary material for: Subinhibitory Concentrations of Antibiotics Exacerbate Staphylococcal Infection by Inducing Bacterial Virulence
Source: Microbiol Spectr. 2022 Jun 27;10(4):e00640-22. doi: 10.1128/spectrum.00640-22 (PMC9431598; doi:10.1128/spectrum.00640-22)
Supplement: Supplemental file 1 — Supplemental material. Download spectrum.00640-22-s0001.pdf, PDF file, 0.4 MB [file spectrum.00640-22-s0001.pdf]

Supplemental information

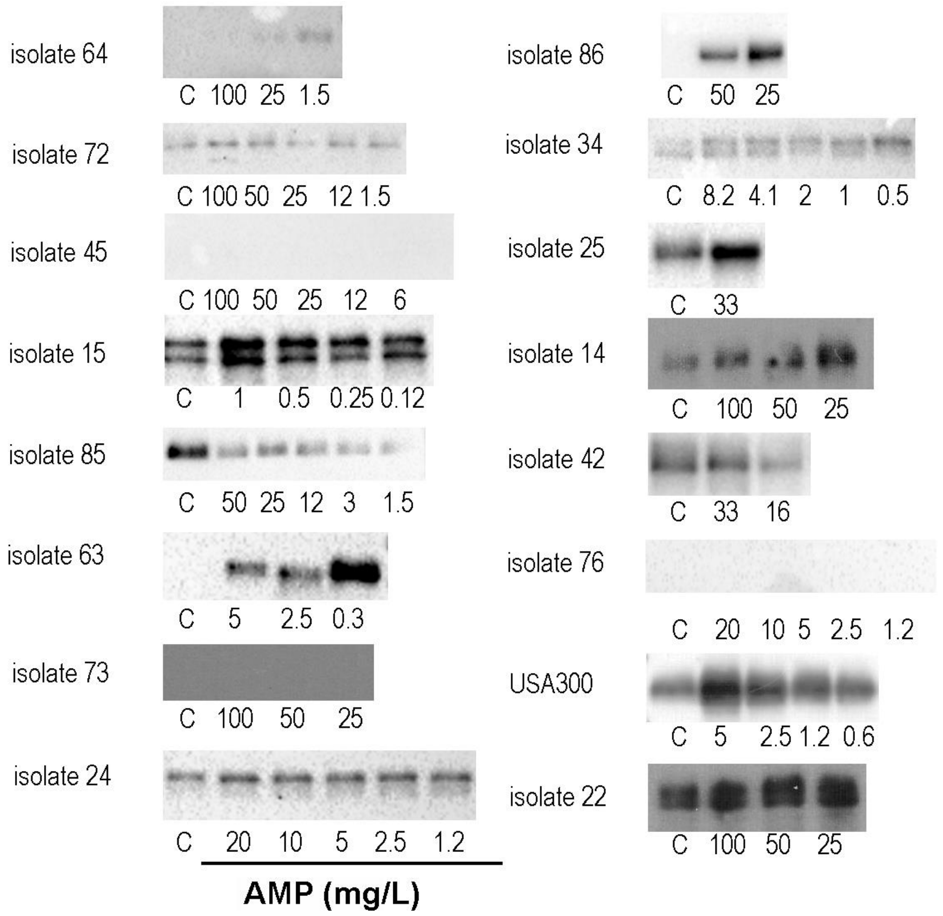

FIG S1 Western blot of protein A at different concentrations of ampicillin in other *S. aureus* isolates. Bacteria were cultured with various concentration of ampicillin for 24h, and protein A production was evaluated by Western blot. USA300 and 15 clinical isolates were tested in this experiment.

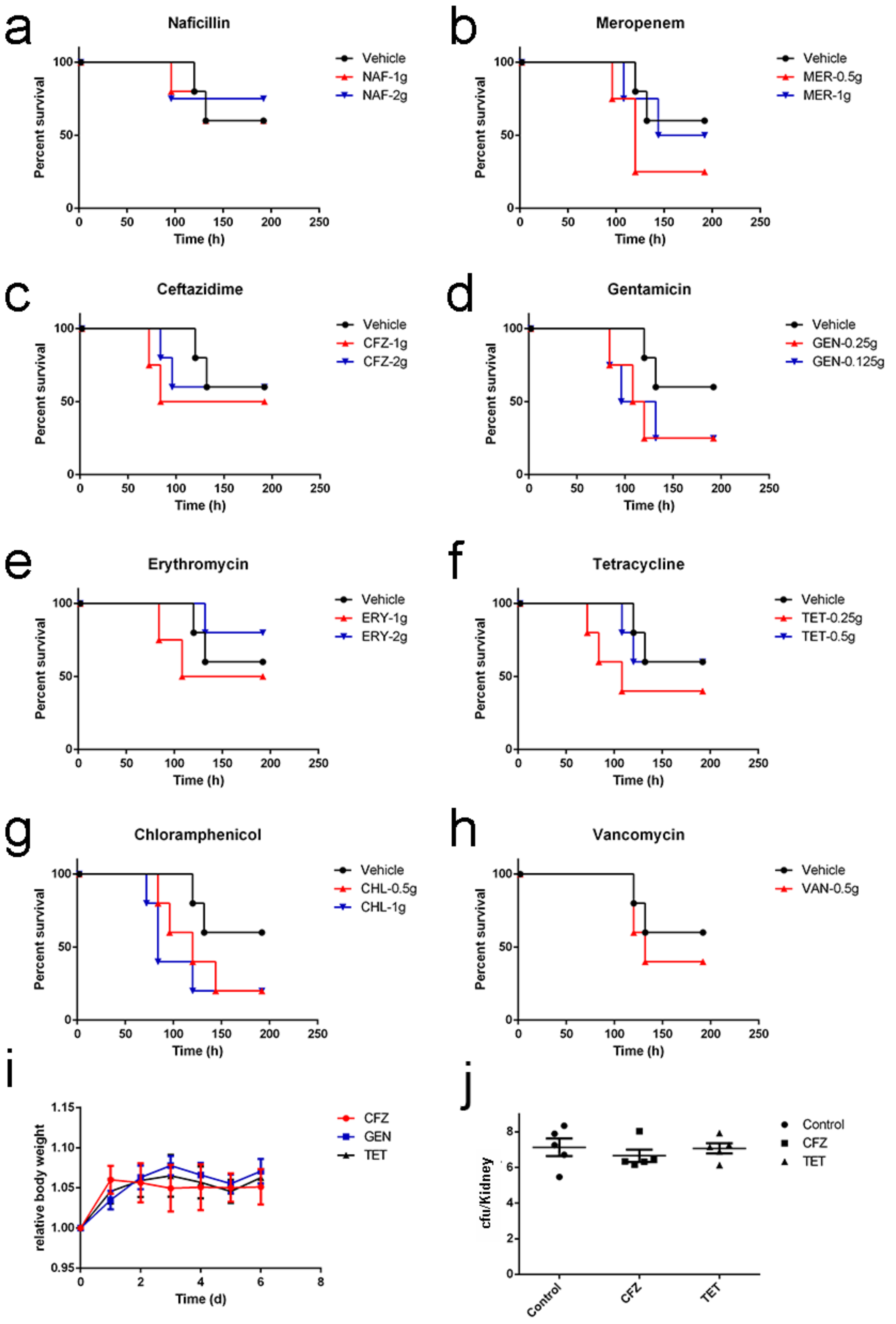

FIG S2 ***S. aureus*** **infected mice treated with eight different antibiotics in a bacteremia model**

(a-h), After infection with Mu3, mice were treated with two dosages of antibiotics or vehicle per day. Dosages corresponding to the human treatment were used in this experiment and listed in table 4. The survival of mice was monitored for 7 days. (i), the mice without infection were treated with CFZ (2g), GEN (0.25g), TET (0.25g) and body weight were monitored for 7 days. (j), Bacterial load were monitored on day 2 after CFZ and TET treatment.
